# Tailoring implementation strategies for scale-up: Preparing to take the Med-South Lifestyle program to scale statewide

**DOI:** 10.3389/frhs.2022.934479

**Published:** 2022-11-23

**Authors:** Jennifer Leeman, Lindy B. Draeger, Kiira Lyons, Lisa Pham, Carmen Samuel-Hodge

**Affiliations:** ^1^School of Nursing, University of North Carolina at Chapel Hill, Chapel Hill, NC, United States; ^2^Center for Health Promotion and Disease Prevention, University of North Carolina at Chapel Hill, Chapel Hill, NC, United States; ^3^Department of Nutrition, Gillings School of Global Public Health, University of North Carolina at Chapel Hill, Chapel Hill, NC, United States

**Keywords:** implementation determinants, implementation strategies, scale-up, function, federally qualified health centers, health departments

## Abstract

**Background:**

Tailoring implementation strategies for scale-up involves engaging stakeholders, identifying implementation determinants, and designing implementation strategies to target those determinants. The purpose of this paper is to describe the multiphase process used to engage stakeholders in tailoring strategies to scale-up the Med-South Lifestyle Program, a research-supported lifestyle behavior change intervention that translates the Mediterranean dietary pattern for the southeastern US.

**Methods:**

Guided by Barker et al. framework, we tailored scale-up strategies over four-phases. In Phase 1, we engaged stakeholders from delivery systems that implement lifestyle interventions and from support systems that provide training and other support for statewide scale-up. In Phase 2, we partnered with delivery systems (community health centers and health departments) to design and pilot test implementation strategies (2014–2019). In Phase 3, we partnered with both delivery and support systems to tailor Phase 2 strategies for scale-up (2019–2021) and are now testing those tailored strategies in a type 3 hybrid study (2021–2023). This paper reports on the Phase 3 methods used to tailor implementation strategies for scale-up. To identify determinants of scale-up, we surveyed North Carolina delivery systems (*n* = 114 community health centers and health departments) and elicited input from delivery and support system stakeholders. We tailored strategies to address identified determinants by adapting the form of Phase 2 strategies while retaining their functions. We pilot tested strategies in three sites and collected data on intermediate, implementation, and effectiveness outcomes.

**Findings:**

Determinants of scale-up included limited staffing, competing priorities, and safety concerns during COVID-19, among others. Tailoring yielded two levels of implementation strategies. At the level of the delivery system, strategies included implementation teams, an implementation blueprint, and cyclical small tests of change. At the level of the support system, strategies included training, educational materials, quality monitoring, and technical assistance. Findings from the pilot study provide evidence for the implementation strategies' reach, acceptability, and feasibility, with mixed findings on fidelity. Strategies were only moderately successful at building delivery system capacity to implement Med-South.

**Conclusions:**

This paper describes the multiphase approach used to plan for Med-South scale-up, including the methods used to tailor two-levels of implementation strategies by identifying and targeting multilevel determinants.

## Introduction

Evidence suggests that implementation strategies are most effective when tailored to address the multilevel factors that determine when an intervention is successfully integrated into practice (1). Multiple researchers have described methods for tailoring implementation strategies to promote and support the implementation of new interventions within one or more settings (2, 3). Fewer have described methods for tailoring implementation strategies to scale-up interventions at the regional or national levels. The purpose of this paper is to describe the process we used to tailor implementation strategies to prepare for statewide scale-up of the Med-South Lifestyle Program.

The Med-South Lifestyle Program (Med-South) is a research-supported intervention with demonstrated effectiveness at improving dietary intake, physical activity, and blood pressure control (4–6). Med-South involves four structured, one-to-one, monthly sessions during which a counselor (health educator, nurse, nutritionist, or community health worker) promotes healthy lifestyle change through education, goal setting, action planning, and referrals to community resources (e.g., places to be physically active). Monthly sessions typically last 45–60 min. Counselors provide shorter 10–15 min booster calls between sessions. In previous studies, counselors have delivered sessions in-person, either in a healthcare setting or the home. In response to COVID-19, counselors in this study delivered the sessions both in-person and *via* phone or videoconference. Formerly called Heart-to-Health, the program has been re-named Med-South to highlight its promotion of a Mediterranean dietary pattern that has been adapted for a southern US population.

We define scale-up as a systematic approach to “rolling out a successful local program to regional, national, or international levels” (7).” In this study, our goal was to move from local to statewide roll out of Med-South. Tailoring implementation strategies for scale-up is different from tailoring strategies to implement an intervention at the local level (8). One reason for this is the need to tailor strategies for each of the two levels of systems involved in scale-up: the delivery system and the support system (9, 10). Delivery systems include the clinical, public health, or other community-based settings that are intended to adopt and implement an intervention into practice. Examples of strategies at the delivery-system level include implementation teams and cyclical, small tests of change (11). Scale-up typically also involves one or more support systems, also referred to as intermediary and purveyor organizations (12, 13), that provide training, technical assistance and other implementation strategies to promote and support delivery systems to adopt and implement an intervention. In addition to requiring strategies at two levels of systems, scale-up often requires tailoring to address determinants beyond those considered during local implementation. For example, strategies will need to be tailored to determinants at the level of the support system (e.g., staffing, resources, mission). Tailoring strategies for scale-up also may require attention to policy, regulatory, budgetary, and other factors that may impede or support uptake at the regional or national level (14).

**Figure 1:**
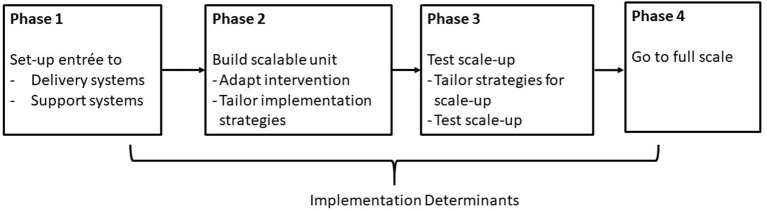
Framework for scaling up interventions, adapted from Barker et al. (15).

In this paper, we describe how we applied an adapted version of Barker et al. (15) framework for scaling up health interventions to prepare for the statewide scale-up of Med-South. We chose Barker's framework because it describes a systematic approach to moving implementation from the local to the regional or national levels. The Barker framework describes scale-up as a four-phase process: (1) set-up entrée, (2) develop the scalable unit, (3) test scale-up, and (4) go to full scale (Figure 1) (15). The purpose of Phase 1 is to engage stakeholders who will provide entrée to two types of organizations: those that will implement the intervention (i.e., delivery systems) and those who will support intervention scale-up (i.e., support systems). Phase 2 involves building the scalable unit or “change package,” which includes the intervention and the implementation strategies needed to put it into practice. Phase 3 involves tailoring implementation strategies to support scale-up and then testing them across multiple settings. In Phase 4, the intervention is taken to scale at the regional or national level. At each phase in the framework, decisions about engaging stakeholders and tailoring strategies are influenced by multilevel barriers and facilitators (i.e., implementation determinants).

The U.S. Centers for Disease Control and Prevention funded this research through two five-year grants to the University of North Carolina's Prevention Research Center. We briefly summarize the first of these studies, during which we completed Phases 1 and 2 of Barker's framework (2014–2019). We then present methods and findings from Phase 3, which we completed during the first 2 years of the second study (2019–2024).

### Phase 1: Set up entrée

To plan for Med-South scale-up, we consulted the Prevention Research Center's community advisory board, which includes representatives from underserved communities, delivery systems, and state-level support systems in North Carolina. With support from the advisory board, we engaged representatives from community health centers (CHC), health departments (HD), and other community organizations (e.g., hospital, wellness center, community college, and agricultural extension) in each of two counties to participate in engaged research/practice workgroups.

### Phase 2: Build scalable unit

The engaged research/practice workgroups adapted the intervention, tailored implementation strategies, and pilot tested both the intervention and implementation strategies in two counties' CHCs and HDs (5, 6, 16). After a 1-year planning period, we conducted two successive, one-arm, type 3 effectiveness-implementation trials; the first trial in the original county and then we replicated Med-South implementation in a second county. Both trials demonstrated broad reach to the intended population, fidelity to intervention protocols, and improvements in participant outcomes (dietary intake, physical activity levels, and blood pressure control) (5, 6). The research team packaged the intervention and implementation strategies into a web-based change package that includes intervention and implementation protocols, a participant handbook, workflows for identifying and referring eligible participants, and metrics for monitoring implementation (https://hpdp.unc.edu/med-south-lifestyle-program/). Table 1 provides an overview of the implementation strategies developed in Phase 2, which are named using terminology from the Expert Recommendations for Implementing Change (ERIC) project (11). For each strategy, the table specifies who enacted the strategy (i.e., research team and/or delivery system). Most strategies were enacted collaboratively by members of both the research team and delivery system. Finally, Table 1 describes each strategy's function (central purpose) and form (the specific activities or formats used to carry out the strategy's central function) (17).

**Table 1:** Phase 2 implementation strategies: Name, actor, function, and form.

**Name (ERIC)^a^**	**Actor**	**Function**	**Form**
Use advisory boards and workgroups	Research team and delivery sysm	Engage users in tailoring, enacting, and improving implementation	Monthly engaged research/practice workgroup meetings to oversee planning, implement a communication plan, review quality monitoring data, and improve implementation
Develop a formal implementation blueprint		Standardize Med-South implementation process	Create workflow diagrams
Develop and distribute educational materials		Provide resources to support intervention delivery	Adapt participant manual, create community resource inventory, and distribute
Conduct ongoing training		Increase delivery system capacity to deliver Med-South per protocols	Deliver five-day, on-site training to counselors and supervisors
Centralize technical assistance	Research team	Monitor and support Med-South delivery per protocols	Make monthly phone calls with counselors to review cases and answer questions
Develop and implement tools for quality monitoring		Monitor Med-South implementation per protocols	Establish tools and protocols to track data on intervention reach, fidelity, and effectiveness

a Strategies are named using terminology from the Expert Recommendations for Implementing Change (ERIC) project (11).

### Phase 3: Testing scale-up

A central product of Phase 2 was the creation of a change package that includes both the intervention and the strategies needed to implement the intervention within CHCs or HDs. The goal of Phase 3 was to develop the strategies needed to take the change package to scale across CHCs and HDs statewide. This required further tailoring of Phase 2 strategies to reduce the high level of research team involvement, which was neither feasible for scale-up nor sustainable over time. Phase 3 also involved the selection and tailoring of new strategies to overcome barriers and leverage facilitators to statewide scale-up.

## Methods

### Design

In Phase 3, we engaged a stakeholder workgroup that included representatives from delivery systems (i.e., CHCs and HDs) and from three of North Carolina's state-level support systems (Institute of Public Health, Area Health Education Centers, and Community Health Center Association). In contrast to Phase 2's highly collaborative research/practice workgroup, the Phase 3 workgroup served in a consultative role to the research team (18). With guidance from our stakeholder workgroup, we conducted formative work to identify multi-level determinants of scale-up and tailor strategies to target those determinants (2019–2020). We then pilot tested strategies using a one-arm pretest/posttest design (2020–2021). The University's Non-Biomedical Institutional Review Board (IRB) approved and monitored the study (#19-2079).

### Setting and sample

Formative data were collected *via* surveys of staff who make decisions about lifestyle programs in all CHCs and HDs in North Carolina and conversations with representatives from state-level support systems. CHC and HD staff were provided a $30 e-gift card for completing surveys. Based on formative findings, implementation strategies were tailored for scale-up strategies and then pilot tested with a convenience sample of three sites (1 CHC, 1 HD, and 1 CHC/HD partnership) that we recruited with input from the stakeholder workgroup. Each site signed a Memorandum of Understanding, in which they committed to identify staff to deliver and implement Med-South, release staff to participate in training and technical assistance, and deliver Med-South to at least 15 patients or clients. Each site was paid $5,000 to reimburse for time spent on study-related activities and $50 for each hour of Med-South delivery. Sites recruited clients/patients to participate in Med-South and then referred them to the research team, who screened for eligibility, obtained informed consent, and collected baseline and follow-up survey data. Eligibility criteria were broad and included anyone over 18 who did not have a health condition requiring them to follow a prescriptive diet (e.g., kidney disease). Med-South participants were reimbursed $40 for each of two data collection phone calls.

### Measures

Table 2 provides an overview of Phase 3 formative and pilot study measures. Further detail on these measures is provided below.

**Table 2:** Pilot study measures.

**Construct**	**Measure**	**Data source**	**Timing**
**Formative**			
Determinants of scale-up [CFIR; (19 )]	Survey	Decision makers at CHCs and HDs	Spring 2020
	Conversations	State-wide support systems	Spring 2020
**Pilot Study**			
Capacity to deliver Med-South	Survey	Staff who implemented Med-South	Completion of training Fall 2020
Capacity to implement Med-South	Survey		Completion of training Fall 2020
Reach (20)	REDCap-based system for tracking enrollment		During recruitment and implementation
			Fall 2020–Spring 2021
Acceptability	WEVAL (21)		Completion of training Fall 2020
			End of study
Feasibility	Focus group interview		Spring 2021
Fidelity (implementation)	Tracking logs		During training
	Structured questions during technical assistance calls		Monthly during implementation
Effectiveness	Survey (dietary intake, physical activity) (22–24)	Clients/patients who participated in Med-South	Baseline and end of study
	Weight		
	Blood Pressure		

#### Formative surveys and discussions

The research team developed an online survey to assess determinants of Med-South scale-up, with input from our stakeholder workgroup. The survey was guided by the Consolidated Framework for Implementation Research [CFIR; (19)] and included a 5-point Likert scale response ranging from strongly disagree to strongly agree. The survey was designed to assess factors relevant to organizational decision-making about Med-South adoption. For this reason, survey items focused on CFIR constructs related to barriers and facilitators at the level of the intervention and the inner and outer settings of the delivery systems where the intervention would be implemented. We administered the survey *via* email to all CHCs and HDs in NC.

To assess the resources available to support scale-up, members of the research team had conversations with leadership from statewide support systems. The research team conducted one-to-one phone or Zoom discussions with leaders of North Carolina's Institute of Public Health, Area Health Education Center, and Association of Community Health Centers. Discussions explored the support each organization was able and willing to provide to recruit CHCs and HDs and provide training or technical assistance to their staff. We also asked our Prevention Research Center advisory board members for input on how we might align Med-South scale-up with other state-level initiatives.

#### Pilot test: Measures of intermediate, implementation, and effectiveness outcomes

**Figure 2:**
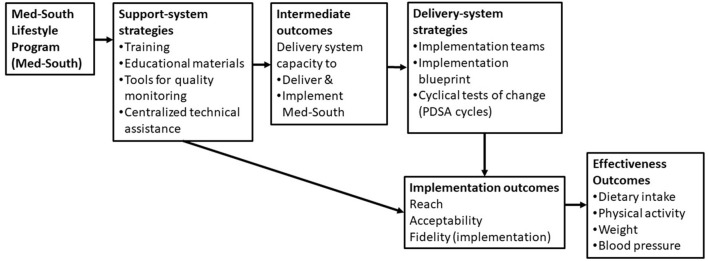
Framework for evaluating Med-South Scale-up.

Figure 2 depicts the framework used to evaluate the pilot test of Med-South scale-up. The figure describes how implementation strategies at the level of the support system were intended to build delivery system capacity to deliver and implement Med-South (intermediate outcomes). The figure further describes how implementation strategies at the level of the delivery system were intended to impact implementation and effectiveness outcomes. Below we describe the measures used to assess each type of outcome.

#### Intermediate outcomes: Capacity to deliver and implement Med-South

Delivery system capacity was operationalized as CHC and HD staff confidence in their ability to deliver and implement Med-South. To assess capacity, we administered two surveys. The first survey assessed confidence to deliver Med-South and included 8 items with a 10-point analog scale (1 = lowest and 10 = highest level of confidence). Items addressed respondents' ability to use Med-South materials (e.g., participant handbook), work with clients to change health behaviors, and share knowledge of nutrition and physical activity guidelines. The second survey included 10 items with a 5-point Likert scale that assessed staff confidence in their ability to work with others to complete implementation strategies (e.g., convene an implementation team). Both surveys were administered at completion of training.

#### Implementation outcomes: Reach, acceptability, feasibility, and fidelity

Reach was operationalized as the number and demographics of clients/patients enrolled and retained (20). Reach data were extracted from REDCap, a secure online system that both the research team and CHC and HD staff used to track data on participant enrollment and participation. We assessed staff perceptions of the acceptability of support system-level strategies (e.g., training) *via* an adapted version of the Workshop Evaluation (WEVAL) survey (21), which was administered at completion of training and assessed perceptions of educational materials and trainings using a five-point Likert response scale. WEVAL is a validated measure administered immediately following training to assess participant perceptions of quality, relevance, and support provided. To further assess acceptability and feasibility, a member of the team with expertise in qualitative methods (JL) conducted three, end-of-study, semi-structured, video-conferenced focus group interviews with the implementation team and counselors at each site. Using an interview guide, staff were asked to reflect on their experience engaging with support system-level strategies (e.g., trainings), applying delivery system-level strategies (e.g., convening an implementation team), and delivering Med-South to their clients/patients. The study project manager monitored fidelity to support-system strategies *via* tracking logs. Fidelity to delivery-system strategies was assessed *via* structured questions during technical assistance calls during which implementation teams were asked to report on progress toward implementing Med-South and revisions made to address those barriers.

#### Effectiveness outcomes

Data were collected to assess blood pressure, weight, and self-reported dietary and physical activity behaviors at baseline and 4-months. Each site's counselors collected blood pressure and weight data at the first and last Med-South counseling sessions. Members of the study team collected data on self-reported dietary behaviors by phone, using a validated brief dietary screener for fruits, vegetables, and fiber (22) and a single item about nuts and nut butter intake from a validated fat quality survey (23). A single item (adapted from the 2 items used in Behavioral Risk Factor Surveillance System [BRFSS]) was used to assess usual daily consumption of sugar-sweetened beverages (24).

### Analysis

Quantitative data were summarized using descriptive statistics. Interviews were audiotaped and transcribed. Two members of the research team applied framework analysis to review interview transcripts to assess perceptions of the acceptability and feasibility of the intervention and implementation strategies, reports of fidelity to implementation strategies, and recommendations for improvement (25). The two team members reviewed transcripts, developed a code book, coded transcripts, and used a matrix to chart key findings across both codes and cases. They met to compare and reconcile coding and matrices. Final analyses were shared with the full research team and stakeholder workgroup.

## Findings

### Formative findings: Determinants of scale-up

Survey respondents (*n* = 114) included 58 HDs and 56 CHCs (67 and 64% response rates, respectively). Table 3 provides an overview of survey responses. A majority of respondents agreed that Med-South had potential to improve lifestyle counseling (82%) and that it would be feasible for staff to deliver the intervention (67%) and attend required trainings (60%). Respondents also agreed that Med South aligned with their organization's mission (85%) and priorities (93%). However, only a minority agreed that Med-South would be feasible for their organization to deliver (40%) or that their organization would have sufficient time (40%) or staff (38%) to implement. Furthermore, only a minority reported that patients/clients would have time (31%) or transportation (13%) to attend sessions or have access to heart healthy foods (18%). Mean scores were not significantly different across geographic region or setting type with one exception: respondents from western North Carolina gave lower ratings to outer-setting level items than respondents in other regions of the state. The COVID-19 pandemic started just prior to survey administration, and in open-ended comments, multiple respondents noted the distinct challenges created by the pandemic.

**Table 3:** Determinants of scale-up: Statewide survey of community health centers and health departments.

**Survey Item**	***n* (%) Agree^a^**	**Mean (SD)**
**Characteristics of the intervention**		
Med-South has potential to improve lifestyle counseling in this organization	89 (82)	4.26 (0.80)
Med-South would be difficult for our staff to deliver	33 (67)^b^	2.97 (0.99)
It would be difficult for our staff to attend training on Med-South	40 (60)^b^	3.09 (1.16)
**Inner setting**		
Promoting healthy eating and physical activity is a priority for our organization	101 (93)	4.61 (0.74)
Med-South fits the mission of our organization	93 (85)	4.39 (0.85)
We have the physical space to implement Med-South	60 (56)	3.46 (1.34)
Implementing the Med-South Lifestyle Program is feasible for our organization	58 (53)	3.57 (1.17)
We have the time to implement Med-South	43 (40)	3.05 (1.17)
We have the staff to implement Med-South	41 (38)	2.94 (1.24)
**Outer setting**		
Our patient/clients want more support to improve lifestyle behaviors	70 (65)	3.80 (0.80)
Our patients/clients would have time to attend Med-South sessions	33 (31)	3.09 (0.79)
Our patients/clients have access to fresh fruits, vegetables, and other heart-healthy foods	19 (18)	2.56 (0.86)
Our patients/clients would have transportation to attend Med-South sessions	14 (13)	2.54 (0.83)

a 4 or 5 on a Likert Scale ranging from 1 = strongly disagree to 5 = strongly agree.

b reverse scored.

Discussions with support system stakeholders also occurred shortly after the start of the COVID-19 pandemic, and stakeholders told us they were overwhelmed with the work required to respond to the pandemic. Concurrently, their resources were heavily invested in preparing CHCs for North Carolina's initiative to transform Medicaid into a medical home model effective July 2020. Nonetheless, two support systems were interested and had some capacity to support scale-up. The North Carolina Area Health Education Centers offered the support of their statewide learning management system, which includes staff and infrastructure to host trainings and process continuing education credits. The North Carolina Institute of Public Health had capacity to support the transition of some training content to an online format. The Prevention Research Center advisory board identified a potential facilitator at the outer setting level: In the near future, the state would be revising its public insurance program (Medicaid) to provide incentives to sites that improve specific quality measures (e.g., control of hemoglobin A1c and high blood pressure).

### Tailoring implementation strategies for scale-up

As summarized in Table 4, tailoring for scale-up was designed to retain the function of Phase 2 strategies and modify their forms. This included transitioning implementation strategies from the research team to either the delivery system (i.e., CHCs and HDs) or an established support system. For example, responsibility for the function “engaging users in tailoring, enacting, and improving implementation strategies” was transitioned from an engaged research/practice workgroup to a site-based implementation team. Based on formative findings, strategies also were tailored to (1) enhance feasibility by reducing burden to CHCs and HDs, (2) limit potential exposure to COVID-19, and (3) leverage support system resources. To reduce burden to CHCs and HDs, we reduced the overall amount of training time and shifted from an in-person to virtual format. To transition trainings to virtual format, we leveraged support system resources to support creation and delivery of both self-directed, online modules and synchronous web-conferences. Converting trainings to a virtual format also served to reduce safety risks during the COVID-19 pandemic. In addition to creating new forms for the functions performed by Phase 2 strategies, we also identified the need to “increase delivery system capacity to implement Med-South per protocols”. This new function was needed to build delivery system capacity to take responsibility for implementation (e.g., create a workflow diagram and implementation plan). The research team also began to plan for ways to leverage the state's new Medicaid transformation initiative to promote and support Med-South as a means of improving quality measures related to control of hemoglobin A1c and high blood pressure.

**Table 4:** Phase 2 strategies tailored for scale-up: strategies, functions, and forms (italics identify new function).

**Phase 2 Strategy (ERIC)^a^**	**Function (new function italicized)**	**Phase 2 Form**	**Phase 3 Strategy (ERIC)^a^**	**Phase 3 Form**
**Strategies transitioned to delivery system**				
Use advisory boards and workgroups	Engage users in tailoring, enacting, and improving implementation	Monthly engaged research/practice workgroup meetings	Organize clinician implementation team meetings	Delivery system leadership designate team members, endorse a team charter and a plan for monthly meetings
Develop a formal implementation blueprint	Standardize Med-South implementation process	Engaged research/practice workgroup creates workflow diagrams	Develop a formal implementation blueprint	Implementation team completes readiness assessment, workflow diagrams, and implementation plan
Centralize technical assistance	Monitor and support Med-South implementation per protocols	Research team makes monthly phone calls to counselors to review cases and answer questions	Conduct cyclical small tests of change	Implementation team conducts Plan-Do-Study-Act (PDSA) cycles to iteratively improve implementation
**Strategies transitioning to support system**				
Conduct ongoing training	Increase delivery system capacity to deliver Med-South per protocols	Research team and members of delivery system deliver five-day, on-site training to counselors and supervisors	Conduct ongoing training (on intervention)	Two 1-h, self-guided online modules on current nutrition and physical activity guidelines & Four 2-h web-conference sessions, with 4 h on Med-South delivery
	*Increase delivery system capacity to implement Med-South*		Provide ongoing training (on implementation)	Four 2-hour web-conference sessions, with 4 hours on Med-South implementation
**Strategies delivered by research team**				
Develop and distribute education materials	Provide resources to support intervention delivery	Adapt participant manual, create community resource inventory, and distribute	Distribute educational materials	Distribute participant manuals and create and distribute community resource inventories
Develop and implement tools for quality monitory	Monitor and support Med-South implementation and delivery per protocols	Establish tools and protocols to track data on intervention reach, fidelity, and effectiveness	Develop and implement tools for quality monitoring	REDCap system used to track Med-South delivery and effectiveness.
Centralize technical assistance		Research team makes monthly phone calls to counselors to review cases and answer questions	Centralize technical assistance	Monthly technical assistance phone calls with implementation teams to review REDCap data and address questions/barriers

CHC, Community Health Center; HD, Health Department.

a Strategies are named using terminology from the Expert Recommendations for Implementing Change (ERIC) project (11).

### Pilot testing scale-up

Figure 2 depicts the framework used to evaluate the pilot test of Med-South scale-up. The figure describes the implementation strategies support systems used to build delivery system capacity to deliver and implement Med-South (intermediate outcomes). The figure further describes the implementation strategies delivery systems used to implement Med-South. Finally, the figure depicts how both support and delivery system strategies impact implementation outcomes, which in turn impact effectiveness outcomes. Below we summarize findings from the pilot test of Med-South scale-up.

### Intermediate outcomes: Capacity to deliver and implement Med-South

At least one counselor at each site (*n* = 5) completed a survey assessing confidence in their ability to deliver and implement Med-South. Means for confidence to deliver Med-South ranged from 7.6 to 8.8 on a 10-point scale, with 10 being highly confident (Table 5). The three items with the lowest mean scores addressed confidence related to nutrition knowledge, physical activity knowledge, and motivational interviewing. Means for confidence to implement Med-South ranged from 4 to 4.6 on a 5-point scale (Table 6). The four items with the lowest mean score addressed confidence related to process flow diagrams and conducting cyclical small tests of change (i.e., Plan-Do-Study-Act cycles).

**Table 5:** Capacity to deliver: Counselor reports of ability to deliver Med-South (*n* = 5).

**Confidence in ability to deliver Med-South intervention** **(*n* = 5; 10-point Analog Scale)**	**Mean**	**Range**
How confident are you that you have what it takes to fulfill your role as a counselor in the Med-South Lifestyle Program?	8.8	8 to 9
How confident are you in your ability to use the Med-South Lifestyle Program participant materials during your sessions and phone contacts?	8.6	7 to 9
How confident do you feel about your ability to lead your participants through the initial 'checking-in' component of each program contact (in-person or by phone)?	8.4	8 to 9
How confident do you feel about your ability to lead your participants through the goal-setting with action-planning component of each session?	8.4	8 to 9
How confident are you in your ability to work with clients to change their lifestyle behaviors?	8.2	7 to 10
How confident do you feel about your knowledge of nutrition for cardiovascular (heart and blood vessel) and chronic disease risk reduction?	7.8	5 to 10
How confident do you feel about your knowledge of physical activity for cardiovascular and chronic disease risk reduction?	7.8	6 to 9
How confident do you feel about using motivational interviewing principles with your Med-South Lifestyle Program participants?	7.6	6 to 9

**Table 6:** Capacity to implement: Counselor reports of ability to implement Med-South (*n* = 5).

**Confidence in ability to work with others in the organization** **(5-point Likert Scale)**	**Mean**	**Range**
Assess my organization's readiness to implement the Med-South Lifestyle Program	4.6	4 to 5
Identify setting-level barriers and facilitators to implementing the Med-South Lifestyle Program	4.6	4 to 5
Convene on a small team to assist in planning and implementing Med-South in my practice setting	4.6	4 to 5
Participate as a member of a small team to assist in planning and implementing Med-South in my practice setting	4.6	4 to 5
Create a Med-South implementation plan	4.6	4 to 5
Adapt Med-South implementation to overcome barriers	4.4	4 to 5
Develop a process flow diagram	4.2	3 to 5
Describe how to use a process diagram to plan for Med-South implementation	4.2	3 to 5
Describe the four steps of a plan-do-study-act cycle	4.2	3 to 5
Conduct small plan-do-study-act cycles to determine the best ways to implement Med-South	4	3 to 5

### Implementation outcomes

#### Reach

The sites enrolled 39 participants of whom 25 completed the 4-month intervention period (Table 7). Completion rates were 100% at Site A, 61.5% at Site B, and 30.8% at Site C (the CHC/HD partnership site), for an overall rate of 64%. Enrollees were predominantly female (79.5%) and Black (60.0%). Of enrolled participants, 28 (72%) attended the first intervention session. For those who attended the first session, 89% (25/28) completed the program and provided follow-up survey data.

**Table 7:** Med-South reach across the three participating sites.

	**Site A**	**Site B**	**Site C**	**Overall**
Enrolled	13	13	13	39
Female	12	10	9	31
Male	1	3	4	8
Black	5	8	10	23
White	8	4	2	14
Other race/ethnicity	0	1	1	2
Completed	13	8	4	25
Female	12	7	2	21
Black	5	5	2	12
White	8	2	1	11
Other	0	1	1	2

#### Acceptability

Seven staff completed the WEVAL survey, and on a five-point Likert scale, all either agreed or strongly agreed that they were satisfied with intervention materials, comfortable using materials, expected to use what they learned in the trainings, and perceived the intervention to be compatible with the needs of their patients/clients. Interview findings provide further support for the acceptability of support system-level implementation strategies. Staff reported that trainings were thorough and the educational materials (i.e., participant handbook) were beautiful. They appreciated the monthly technical assistance calls and the research team's responsiveness to questions during calls and *via* email. Staff also identified concerns with the support system strategies, including the gap between completion of training and the first counseling session and difficulties with the electronic REDCap system used to capture data on intervention delivery. Staff were highly satisfied with the Med-South intervention which they viewed as an opportunity to improve patients' health and to try something new.

#### Implementation fidelity

Fidelity to support system strategies was high. Trainings and technical assistance were delivered as intended, with high levels of participation from CHC and HD staff. Fidelity to delivery system implementation strategies was mixed. All three sites held monthly implementation meetings, used process flow diagrams, and communicated with key stakeholders. Only two of three sites used implementation plans, and none of the sites used readiness assessments or PDSA cycles as intended.

#### Feasibility

Staff identified several barriers to implementing and delivering Med-South. Sites had limited staff to deliver Med-South, particularly during the COVID-19 pandemic, and staff had multiple competing demands on their time. Patients and clients also had limited time available to schedule 1-h counseling sessions, especially during the workday. At the one site where a CHC and HD partnered on implementation, HD staff had difficulty engaging participants who were referred by the CHC, and therefore unfamiliar with the HD.

#### Effectiveness outcomes

Sample sizes were not sufficient to test for statistical significance. On average sites observed clinically meaningful improvements in blood pressure levels, and mean improvements in dietary behaviors and weight changes were similar to those observed in trials of the Med-South program (5, 6); (Table 8).

**Table 8:** Change in effectiveness outcomes: baseline to 4-months.

**Site (Program completers)**	**Daily servings of fruits and vegetables**	**Weight (lb.)**	**Systolic blood pressure (mm Hg)**	**Diastolic blood pressure (mm Hg)**
HD (*n* = 13)	+0.4	−2.4	−3.5	−0.6
CHC (*n* = 8)	+0.9	+0.07	−4.6	−3.2
HD/CHC (*n* = 4)	+1.3	+0.1	−8.3	−5.2
Average Pre-/Post-Change	+0.9	−2.2	−5.5	−3.0

## Discussion

In this paper, we illustrate the use of a multiphase process to tailor implementation strategies in preparation for scale-up. The process followed Barker and colleagues' four-phase framework which, similar to other scale-up frameworks, describes multiple phases that begin with formative work and progress from small pilot studies to full scale implementation (26). We contribute to these prior frameworks by distinguishing two levels of implementation strategies and by describing the process used to tailor strategies for scale-up across phases. In describing the tailoring process, we specify how we largely retained the strategies' functions across phases and tailored their forms to (a) transition strategies from the research team to delivery or support systems and (b) address barriers to implementation and scale-up.

Study findings provide initial support for the feasibility, acceptability, and impact of the strategies used to scale-up Med-South. At the height of the COVID-19 pandemic, the three pilot sites reached 39 patients/clients and retained 25, almost half of whom were African Americans. Most drop out occurred after completing the baseline survey and prior to participating in the first intervention session. For patients/clients who attended the first session, 89% completed the program. Of note, completion rates varied across the three sites with the CHC/HD partnership site retaining only 4 of the 13 patients enrolled. CHC and HD staff reported moderate to high levels of confidence in their ability to deliver and implement Med-South, viewed implementation strategies as acceptable, and used some of the implementation strategies with fidelity to protocols. These findings are based on small sample sizes with the goal of contributing to the progressive refinement of strategies over iterative cycles of testing.

Our ability to fully transition implementation strategies to established support systems was limited by the COVID-19 pandemic. During the study period, both delivery and support systems were overwhelmed by the demands of vaccination and testing. Following an initial delay in recruitment, we were able to identify HDs and CHCs whose staff were ready to focus their attention beyond COVID-19. This was less true for support systems who experienced a continued need to focus their resources on supporting delivery system response to the pandemic. Nevertheless, we identified two support systems that were willing to assume some responsibility for training and plan to explore opportunities to further transition responsibility to support systems as we move forward.

The multiphase process used in this study is applicable to scale-up initiatives involving a range of delivery systems, for example, hospitals, clinics, health departments, and schools, among others. This study's process also is applicable to a range of support systems, including any government, academic, for-profit, or non-profit organizations that provides support to delivery systems (8). In this study, support system-level strategies focused on horizontal scale-up or the spread of Med-South across settings. Future research is needed to develop and test support system strategies that focus on vertical scale-up, in other words, on strategies that target change at the “policy, political, legal, regulatory, budgetary or other health systems changes” needed to support an intervention's scale-up at the regional or national level [(14), p. 21].

The multiphase process used in this study is particularly relevant when researchers function as the primary support system for the initial implementation and scale-up of new interventions. The extent of researcher engagement varies. In this study and many others, researchers initially developed a highly engaged, collaborative relationship with community and practice partners to co-create implementation strategies. This initial high-level of engagement provided an in-depth understanding of implementation determinants in the local setting and the strategies needed to address them (18). This high-level of researcher engagement is difficult to scale-up and sustain across many practice settings (26, 27). We describe how we addressed this challenge by reducing the research team's engagement in implementation and transitioning responsibility for implementation to either the delivery system or an established support system. One of the central goals of this transition was to build delivery-system capacity to assume responsibility for the functions previously performed by the highly-engaged research/practice workgroup. Specifically, we created site-based implementation teams and trained them to tailor, enact, and iteratively improve implementation strategies. As detailed below, we were only partially successful in achieving this objective.

As depicted in this study's evaluation framework (Figure 1), the success of Med-South scale-up was contingent on building delivery system capacity to both deliver and implement Med-South. Findings from the pilot study indicate that staff were confident in their ability to deliver Med-South. Findings on staff capacity to implement Med-South were mixed, including only moderate levels of confidence and low fidelity related to creating process follow diagrams and conducting cyclical, small tests of change (i.e., PDSA cycles), two methods delivery systems can use to tailor implementation strategies. Delivery system capacity to tailor implementation to local needs is critical to implementing and sustaining intervention. Delivery systems need to have the capacity to map processes, identify barriers, and monitor and address gaps in implementation so they can tailor implementation to the needs of their community and context (e.g., staffing, population served, funding) (28). The challenges this study experienced are not unique. Multiple researchers have reported on delivery systems' low levels of adherence to PDSA cycle protocols (29). This study is novel in providing the data needed to advance understanding of where gaps occurred, including data on implementation outcomes at the levels of both support and delivery systems as well as intermediate outcomes to assess whether strategies had the intended effects on implementation determinants (e.g., delivery system capacity). Using a multiphase approach to scale-up has allowed us to tailor strategies to address gaps and iteratively prepare for scale-up. The gap in delivery system capacity may have resulted, in part, from how training was tailored to a shorter, virtual format. To address this gap, we have further tailored protocols for technical assistance to reinforce training content related to implementation and to require sites to report back on the completion of specific implementation activities (e.g., completing PDSA cycles). We are testing the revised strategies in our current study of Med-South scale-up across 20 HDs and CHCs (2021–2023).

## Conclusions

This paper illustrates how a multiphase approach was used to prepare for the statewide scale-up of a health intervention, with a specific focus on tailoring two-levels of implementation strategies. The approach might be applied to plan for the scale-up of interventions across a range of delivery and support systems.

## Data availability statement

The raw data supporting the conclusions of this article will be made available by the authors, without undue reservation.

## Ethics statement

The studies involving human participants were reviewed and approved by the University of North Carolina at Chapel Hill's Non-biomedical Institutional Review Board. Written informed consent for participation was not required for this study in accordance with the national legislation and the institutional requirements.

## Author contributions

JL and CS-H led the research study. JL drafted the manuscript. All authors participated in data collection and analysis, contributed to the interpretation of findings and tailoring of implementation strategies, and provided feedback on the manuscript.

## Funding

This publication was funded by the Centers for Disease Control and Prevention of the U.S. Department of Health and Human Services (HHS) as part of a financial assistance award (cooperative agreement numbers: U48 DP006400) with 100 percent funded by CDC/HHS. The contents are those of the author(s) and do not necessarily represent the official views of, nor an endorsement, by CDC/HHS, or the U.S. Government.

## Conflict of interest

The authors declare that the research was conducted in the absence of any commercial or financial relationships that could be construed as a potential conflict of interest.

## Publisher's note

All claims expressed in this article are solely those of the authors and do not necessarily represent those of their affiliated organizations, or those of the publisher, the editors and the reviewers. Any product that may be evaluated in this article, or claim that may be made by its manufacturer, is not guaranteed or endorsed by the publisher.
